# Seasonal runoff-driven transport of oxytetracycline from swine feces and its effects on soil bacteria and antimicrobial resistance risk

**DOI:** 10.3389/fvets.2026.1919739

**Published:** 2026-10-12

**Authors:** Joaquín Mozo, Julieta María Decundo, Guadalupe Martínez, Susana Nelly Dieguez, Alejandro Luis Soraci, Denisa Soledad Pérez Gaudio

**Affiliations:** 1 Laboratorio de Toxicología, Departamento de Fisiopatología, CIVETAN, Facultad de Ciencias Veterinarias, Universidad Nacional del Centro de la Provincia de Buenos Aires (UNCPBA), Tandil, Buenos Aires, Argentina; 2 Departamento de Producción Animal, Facultad de Ciencias Veterinarias, UNCPBA, Tandil, Buenos Aires, Argentina; 3 Consejo Nacional de Investigaciones Científicas y Técnicas (CONICET), Buenos Aires, Argentina; 4 Comisión de Investigaciones Científicas de la Provincia de Buenos Aires (CICPBA), La Plata, Buenos Aires, Argentina

**Keywords:** oxytetracycline, PNEC_MIC_, risk quotient, runoff transport, seasonality, soil bacteria, swine manure

## Abstract

**Inroduction:**

The environmental dissemination of veterinary antibiotics through livestock feces represents a relevant concern within the One Health framework due to its potential effects on ecosystem functioning and antimicrobial resistance (AMR) selection. Oxytetracycline (OTC), widely used in swine production systems, is excreted largely as parent compound and may reach surrounding environments through rainfall-driven runoff. This study evaluated the seasonal persistence and runoff-mediated transport of OTC from swine feces under natural field conditions.

**Methods:**

Experimental fecal deposits fortified with OTC were exposed outdoors for 30 days across four seasons, and runoff water generated after natural precipitation events was collected and analyzed by HPLC-UV. In parallel, culturable aerobic mesophilic bacteria were quantified in exposed soils to assess potential microbiological responses. Meteorological variables were integrated to evaluate their influence on OTC mobilization and environmental behavior.

**Results and discussion:**

OTC residues were detected in runoff water in all seasons and persisted until late-stage rainfall events, indicating sustained environmental availability and continuous hydrological transport. Higher concentrations were generally associated with increased precipitation intensity and erosivity, whereas cumulative precipitation and temperature emerged as key explanatory variables in regression models. Sediment production followed similar seasonal patterns, highlighting the potential relevance of particle-associated transport pathways that warrant further quantitative investigation. OTC-exposed soils showed significantly higher culturable bacterial counts than controls, indicating shifts in microbial abundance under antibiotic exposure, which potentially reflects the selection of tolerant bacterial populations. Importantly, all detected concentrations exceeded the predicted no-effect concentration for resistance selection (PNEC_MIC_), resulting in consistently high risk quotients across all seasons, and indicating a high potential risk for resistance selection. These findings demonstrate that runoff represents a relevant pathway for the environmental dissemination of OTC from swine feces under realistic field conditions. The persistence of residues and the consistently high AMR selection risk highlight the need to integrate environmental monitoring and resistance risk assessment into livestock waste management strategies.

## 1 Introduction

The intensive use of veterinary antimicrobials in livestock production systems has generated growing concern regarding their environmental dissemination and potential contribution to antimicrobial resistance development. Since the discovery of antibiotics during the 1940s, these compounds have played a fundamental role in both human and veterinary medicine by improving disease control, animal welfare, and productive efficiency (1, 2). However, the extensive use of antimicrobials in food-producing animals has also increased the release of pharmaceutical residues into environmental compartments, particularly soil and water systems (3). A substantial proportion of administered antibiotics is not completely absorbed or metabolized and is therefore excreted through feces and urine as parent compounds or biologically active metabolites (73, 4)), facilitating their dissemination into the environment through manure deposition, wastewater, and agricultural land application practices (5).

In intensive swine production systems, antimicrobials occupy a central role in therapeutic, metaphylactic, and, in some regions, prophylactic management strategies (6). The widespread administration of antibiotics under high-density production conditions has raised concerns regarding the environmental accumulation of antimicrobial residues and the associated selection of resistant bacterial populations (7). Animal manure and fecal waste originating from medicated livestock are frequently stored in lagoons, incorporated into agricultural soils, or applied through fertigation practices, creating multiple pathways for environmental contamination. Residual antibiotics present in these matrices may subsequently migrate into adjacent ecosystems through infiltration, leaching, sediment transport, and surface runoff processes (8, 9).

Among veterinary antimicrobials, tetracyclines constitute one of the most extensively used antibiotic classes worldwide due to their broad antimicrobial spectrum, low cost, and therapeutic versatility in livestock production (10–12). Oxytetracycline (OTC), a first-generation tetracycline originally isolated from *Streptomyces rimosus* (74), remains widely employed in swine production systems for therapeutic and metaphylactic purposes, commonly administered at oral doses ranging from 20 to 40 mg/kg body weight depending on the formulation and treatment protocol (13, 14). Although OTC can be administered through parenteral or oral routes, oral administration remains extensively used in intensive swine production because it facilitates mass medication through feed or drinking water and reduces handling-associated stress and labor requirements. However, oral bioavailability in pigs is generally low and strongly influenced by feed composition and the presence of divalent cations in water or diet (13). Consequently, a substantial proportion of the administered drug remains unabsorbed within the gastrointestinal tract and may be excreted through feces (15), facilitating environmental exposure following manure disposal or land application practices.

The environmental behavior of OTC is strongly influenced by its physicochemical properties. Tetracyclines exhibit high affinity for soil particles, organic matter, and multivalent cations, favoring adsorption within soil-feces matrices and sediment compartments (16, 17). Although adsorption may reduce immediate mobility, it may simultaneously prolong environmental persistence by maintaining environmentally available residue pools over time (3). Previous reviews have documented the widespread occurrence of tetracycline residues in agricultural soils, surface waters, groundwater, sediments, and drinking water sources associated with livestock production and agricultural activities worldwide (18–20). Consequently, precipitation and runoff events may mobilize both dissolved and particle-associated OTC fractions into adjacent aquatic environments, particularly under conditions of elevated rainfall intensity and erosive dynamics.

Several studies have reported the occurrence of tetracycline residues in agricultural soils, livestock manure, sediments, and aquatic systems associated with animal production activities (21, 22). Concentrations of OTC in swine manure exceeding hundreds of mg kg^−1^ have been documented in intensive livestock regions (23), highlighting the potential magnitude of environmental release associated with veterinary antibiotic use. OTC has also been detected in agricultural runoff waters, irrigation return flows, rivers, aquaculture environments, and vegetable-production soils impacted by manure application practices (24–26). However, comparatively limited attention has been devoted to evaluating the dissemination of manure-associated antibiotics into aquatic environments through runoff processes occurring after natural rainfall events. Surface runoff represents a particularly relevant pathway for contaminant transport because precipitation may mobilize both dissolved and particle-associated antibiotic fractions from fecal deposits and soil surfaces into adjacent water bodies. Field studies have demonstrated that only a relatively small fraction of manure-associated tetracyclines is typically mobilized through runoff processes, generally representing less than 0.1–0.13% of the initially applied mass, although these concentrations may still remain environmentally relevant due to the high antimicrobial loads present in livestock wastes (25, 27). Despite the recognized environmental relevance of these processes, only a limited number of studies have evaluated antibiotic migration from agricultural soils into surface waters under environmentally realistic field conditions integrating seasonal climatic variability, runoff dynamics, sediment mobilization, and microbial responses (28).

The mobilization of veterinary antibiotics through runoff is influenced by the interaction among multiple environmental variables, including precipitation amount, rainfall intensity, erosivity, soil moisture, temperature, and solar radiation (3). These factors may affect antibiotic persistence, desorption, and hydrological transport within soil-feces matrices under field conditions.

Rainfall intensity and erosivity are recognized as major drivers of runoff-associated contaminant transport because they influence surface flow generation, sediment mobilization, and particulate displacement processes. In this context, the maximum 30-min rainfall intensity (I30) and the rainfall erosivity index (E·I30) are widely used hydrological indicators for estimating the erosive potential of precipitation events and their capacity to mobilize soil-associated contaminants (29, 30).

Due to the strong adsorption affinity of tetracyclines for soil particles and organic matter (16), runoff-associated dissemination may involve both dissolved and particulate fractions, particularly during erosive precipitation events. However, despite the recognized importance of these environmental processes, few field studies have simultaneously integrated seasonal climatic variability, natural precipitation events, runoff-associated antibiotic transport, sediment mobilization, bacterial responses, and resistance-selection risk assessment within environmentally realistic livestock exposure scenarios.

Beyond their occurrence in environmental compartments, residual antibiotic concentrations may exert selective pressure on environmental microbial communities even at subinhibitory concentrations. Increasing evidence indicates that antibiotic concentrations below minimal inhibitory concentrations may promote the enrichment of resistant bacterial populations, facilitate horizontal gene transfer, and alter microbial community structure within environmental ecosystems (31–33). Consequently, the environmental dimension of antimicrobial resistance has become an important component of the One Health framework linking animal, human, and environmental health (34–36).

In recent years, predicted no-effect concentrations associated with antimicrobial resistance selection (PNEC_MIC_) have been proposed as environmentally relevant thresholds for evaluating the selective risk posed by antibiotic residues in environmental matrices (37). These thresholds are derived from minimum inhibitory concentration distributions and represent concentrations below which resistance selection is not expected to occur within environmental bacterial communities. Importantly, several studies have demonstrated that environmental antibiotic concentrations may exceed these thresholds, particularly in environments impacted by livestock production or pharmaceutical contamination (38, 39). In parallel, risk quotient (RQ) approaches comparing measured environmental concentrations with PNEC thresholds have emerged as useful tools for estimating the ecological relevance and resistance-selection potential associated with environmental antibiotic exposure (37, 40).

Although previous investigations have evaluated tetracycline occurrence in soils and aquatic systems, information remains limited regarding the integrated assessment of OTC persistence, runoff-associated transport, sediment mobilization, meteorological variability, and soil bacterial responses under natural field exposure conditions. Moreover, most available studies have been conducted under controlled laboratory conditions or have focused on isolated environmental compartments without incorporating seasonal climatic dynamics.

Therefore, the aims of the present study were: (i) to evaluate the occurrence and persistence of OTC residues in runoff water derived from swine feces exposed under natural environmental conditions; (ii) to determine the abundance of culturable aerobic mesophilic bacteria in exposed soils; (iii) to analyze the influence of meteorological variables on OTC environmental transport and persistence; and (iv) to evaluate the theoretical potential for antimicrobial resistance selection posed by detected concentrations using a PNEC_MIC_-based risk assessment approach.

## 2 Materials and methods

### 2.1 Study area and experimental design

The study was conducted at the experimental facilities of the Centro de Investigación Veterinaria de Tandil (CIVETAN), Faculty of Veterinary Sciences, National University of the Center of Buenos Aires Province (UNCPBA), Tandil, Buenos Aires, Argentina.

Experimental assays were performed outdoors on natural grass-covered soil plots with slight slope conditions allowing surface runoff generation following natural rainfall events. The experiment was repeated during summer, autumn, winter, and spring in order to evaluate seasonal variations in OTC environmental behavior under natural environmental conditions.

Fresh fecal samples were obtained from clinically healthy pigs not receiving antimicrobial treatment. Experimental fecal deposits consisted of 100 g of fresh fecal material placed directly onto the soil surface. To simulate environmentally relevant OTC excretion under therapeutic conditions, fecal samples were experimentally fortified considering the upper therapeutic oral dose commonly used in swine production (40 mg/kg body weight) and the oral bioavailability previously reported for pigs (~6.13%) (13). The final fortification level was established based on the estimated non-absorbed fraction of OTC potentially available for fecal excretion following oral administration.

Non-fortified fecal deposits were included as negative controls in order to obtain blank runoff water and control soil samples under identical environmental exposure conditions. Fecal deposits were established in triplicate per treatment, and maintained outdoors under natural environmental conditions for 30 consecutive days during each seasonal assay.

### 2.2 Runoff water collection

Runoff water samples were collected after natural precipitation events using passive runoff collectors positioned downslope from each fecal deposition point. Collectors were designed to recover surface runoff without interfering with natural flow dynamics.

Following each rainfall event occurring during the exposure period, runoff samples were transferred into sterile polypropylene containers and transported under refrigerated conditions to the laboratory where they were immediately stored at −20 °C in the dark to preserve analyte stability. Runoff water collected from non-fortified fecal treatments was used as blank matrix for chromatographic calibration and analytical quality control procedures. The sampling schedule and number of runoff events varied according to seasonal precipitation patterns.

### 2.3 Sediment collection

The remaining runoff water from successive precipitation events within each seasonal assay was pooled in larger storage containers associated with each passive collector, allowing suspended particulate material to settle naturally over time.

At the end of each seasonal experimental period, the accumulated sediment fraction was recovered by carefully removing the supernatant water in order to minimize particulate loss. Sediment accumulation associated with runoff transport was estimated through a gravimetric recovery procedure based on natural settling of suspended particulate material during the seasonal experimental period.

Recovered sediments were subjected to gravimetric determination by drying at 60 °C until constant weight (41–43), allowing estimation of the total sediment mass generated during each seasonal assay.

### 2.4 Soil sampling and bacterial counts

All microbiological procedures were conducted according to standardized protocols for environmental soil microbiology.

At the end of each 30-day exposure period, superficial soil samples were collected beneath each fecal deposition site for microbiological analysis. Five subsamples per treatment were obtained from the 0–10 cm soil layer using an agricultural core sampler (soil auger) and homogenized to generate a composite sample representative of each experimental condition. Control soils corresponded to areas underlying non-fortified fecal deposits exposed under identical environmental conditions. This control design was specifically selected to isolate the unique ecological impact of the antibiotic residue by standardizing the background nutrient and microbial inputs provided by the fecal matrix across all experimental units. Collected samples were transported to the laboratory under refrigerated conditions and processed within 24 h after collection.

Culturable aerobic mesophilic bacteria were quantified using conventional culture-based methodologies. Briefly, serial decimal dilutions were prepared in sterile diluent solution and inoculated onto nutrient agar using the pour plate technique. Plates were incubated aerobically at 28 °C for 24–48 h, after which bacterial counts were determined and expressed as colony-forming units per gram of dry soil (CFU g^−1^), following standard microbiological procedures for soil analysis (44–46).

### 2.5 Determination of OTC concentrations in runoff water

Residual OTC concentrations in runoff water were determined by high-performance liquid chromatography coupled to ultraviolet detection (HPLC-UV).

Prior to chromatographic analysis, stored runoff water samples were thawed and processed in batches at the end of the 30-day experimental period corresponding to each respective season. Samples were centrifuged at 14,000 rpm for 5 min in 1.5 mL polypropylene microtubes in order to promote particulate sedimentation. The supernatants were subsequently filtered through 0.22 μm nylon membrane filters directly into chromatographic vials before HPLC-UV analysis.

Chromatographic analyses were performed using a Gilson HPLC system equipped with a UV–Vis detector and an autosampler (Thermo Scientific UltiMate 3,000). Separation was achieved using a Luna C18 column (5 μm, 250 × 4.6 mm; Phenomenex) maintained at 40 °C.

The mobile phase consisted of acetonitrile:methanol (50:50) and 0.01 M oxalic acid aqueous solution (50:50), operating under isocratic conditions at a flow rate of 1.5 mL/min. Detection was performed at 365 nm, with an injection volume of 50 μL and a total run time of 5 min. The retention time of OTC was 2.07 ± 0.03 min.

Quantification was conducted using matrix-matched calibration curves prepared in filtered blank runoff/rainwater samples collected from the experimental area in the absence of antibiotic exposure. Calibration standards ranged from 0.06 to 8.00 μg/mL.

Validation parameters were established in accordance with international guidelines (47). The method demonstrated high analytical robusticity, showing excellent selectivity and strict linearity within the evaluated concentration range from the limit of quantification (LOQ) of 0.06 up to 8.00 μg/mL (r^2^ > 0.999 for all replicates). Method accuracy and precision were evaluated using spiked matrix samples at three quality control levels (0.06, 1.00, and 8.00 μg/mL). Accuracy, expressed as relative error, was −3.56%, −1.76%, and −3.02%, respectively. Method precision, expressed as the coefficient of variation (CV%), was consistently below 10% across all evaluated concentrations, confirming robust measurement reliability even at the lowest quantifiable levels.

### 2.6 Meteorological characterization

Meteorological data were obtained from an automated agrometeorological station operated by the Remote Sensing Group of the Instituto de Hidrología de Llanuras (GT-IHLLA) and located at the UNCPBA campus, in close proximity to the experimental site. The station continuously recorded environmental variables including air temperature, relative air humidity, accumulated precipitation, superficial and lower soil temperature, soil volumetric water content, soil electrical conductivity, soil heat flux, atmospheric pressure, and precipitation dynamics. Due to incomplete radiation records from the local station during part of the experimental period, global solar radiation data were complemented using information from a nearby meteorological station within the same monitoring network.

Based on the primary meteorological records, additional environmental variables associated with runoff generation and erosive processes were subsequently calculated for each rainfall event and experimental period. Consecutive precipitation periods were considered independent rainfall events when separated by a minimum dry interval of 6 h without recorded precipitation, following criteria commonly adopted in rainfall erosivity studies (30, 48). This criterion was used to define the number of rainfall events per day and to calculate event-associated meteorological variables.

Derived variables included event day (0–30), the number of rainfall events per day, daily precipitation, cumulative precipitation up to each sampling event, maximum and minimum air temperature, cumulative air temperature, mean superficial and lower soil temperatures, mean daily relative humidity, mean relative humidity during the assay, maximum and mean accumulated radiation, and mean soil moisture during precipitation events and throughout the assay. In addition, rainfall erosivity variables associated with runoff and sediment mobilization processes were calculated for each precipitation event, including I30, maximum incremental intensity, and the E·I30 rainfall erosivity index, where E represents rainfall kinetic energy and I30 corresponds to the maximum 30-min rainfall intensity within each event (30, 49).

Meteorological information was integrated into the analysis to evaluate the influence of climatic conditions on OTC persistence in fecal material, transport into runoff water, and soil bacterial responses.

### 2.7 Statistical analysis

All statistical analyses were performed using RStudio software version 4.5.0. Seasonal differences in meteorological and hydrological variables were evaluated using the Kruskal-Wallis test, considering individual rainfall events as observational units. For variables derived from rainfall events, values recorded during each event were compared among seasons. OTC concentrations in runoff water were compared among seasons using the Kruskal-Wallis test applied to all individual measurements (three samplers × sampling events per season). When the Kruskal-Wallis test indicated statistically significant differences (*p* < 0.05), post-hoc comparisons among seasons were performed using Dunn’s test with Benjamini-Hochberg correction.

Prior to correlation analysis, normality of variables was assessed using the Shapiro–Wilk test. Since most variables deviated significantly from normality (*p* < 0.05), associations between OTC concentrations in runoff water and meteorological and hydrological variables were evaluated using Spearman’s rank correlation coefficient. A correlation matrix was constructed including all relevant meteorological and hydrological variables, and significant correlations (*p* < 0.05) are indicated in the figure.

A two-way ANOVA was performed to assess the effects of treatment (control or OTC treatment), season, and their interaction on edaphic bacterial counts. When significant effects were observed, among groups were identified using Tukey’s post-hoc test.

Multiple linear regression models were developed to evaluate the combined influence of meteorological variables on OTC concentrations in runoff water and on edaphic bacterial counts. Model selection was conducted according to the corrected Akaike Information Criterion (AICc), considering the most parsimonious models with the best explanatory performance.

#### 2.7.1 Environmental risk assessment and resistance-selection analysis

Measured environmental concentrations (MECs) of OTC detected in runoff water were interpreted in the context of potential antimicrobial resistance selection pressure.

Risk characterization was performed by comparing detected environmental concentrations with predicted no-effect concentrations associated with resistance selection (PNEC_MIC_).

Risk quotients (RQ) were calculated as follows:

$$\mathrm{RQ}=\mathrm{MEC}/({\text{PNEC}}_{\mathrm{MIC}})$$

where MEC corresponds to the measured environmental concentration of OTC in runoff water and PNEC_MIC_ represents the threshold concentration below which resistance selection is not expected to occur.

The PNEC_MIC_ value adopted for OTC was 0.5 μg/L, according to previously proposed environmental thresholds for resistance selection in aquatic environments (37, 50). Importantly, this threshold represents a compound-specific predicted value for oxytetracycline rather than a generic or group-level threshold for the tetracycline class. This value was originally derived by Bengtsson-Palme and Larsson (37) by modeling minimal inhibitory concentration (MIC) distributions from the European Committee on Antimicrobial Susceptibility Testing (EUCAST) database to identify the lowest selective boundaries for specific target pharmaceuticals, and it is currently maintained as the validated standard target discharge concentration for oxytetracycline by the AMR Industry Alliance.

Risk quotients were interpreted as indicators of low risk when RQ values were below 0.1, moderate risk when values ranged between 0.1 and 1, and high risk for antimicrobial resistance selection when RQ values were equal to or greater than 1 (37).

Risk assessment was performed for each precipitation event in which OTC concentrations were detected in runoff water in order to estimate the potential selective pressure exerted by antibiotic residues under natural environmental conditions.

## 3 Results

### 3.1 Meteorological characterization of seasonal assays

Marked seasonal differences were observed in the meteorological conditions recorded during the experimental periods. Table 1 presents seasonal summary statistics (mean ± SD) for the meteorological and hydrological variables measured during the study, whereas Supplementary Table S1 provides the corresponding event-level measurements recorded throughout the experimental period.

**Table 1:** Seasonal meteorological conditions, rainfall characteristics, erosivity parameters, soil moisture, relative humidity, and solar radiation recorded during the seasonal assays evaluating oxytetracycline (OTC) mobilization under natural environmental conditions.

Variables	Season				*p*-values
	Summer	Autumn	Winter	Spring	
Event days (0–30)	2, 5, 7, 9, 11, 12, 13, 14, 17, 30	23, 30	1, 11, 21, 22	4, 8, 9, 11, 21	
Daily precipitation (mm)	18.05 ± 12.475a	1.3 ± 0.14b	5.55 ± 5.25b	10.68 ± 11.04ab	*p* = 0.043
Cumulative precipitation (mm)	119.99 ± 56.64a	4.30 ± 0.99b	18.65 ± 5.05b	35.20 ± 23.67b	*p* = 0.002
I30 (mm)	7.87 ± 5.02a	0.60 ± 0.56b	0.95 ± 0.30b	2.12 ± 3.22b	*p* = 0.006
Maximum incremental intensity (mm min^−1^)	0.29 ± 0.68	0.11 ± 0.11	0.05 ± 0.03	0.03 ± 0.02	*p* = 0.752
E·I30 (MJ.mm/ha.h)	3.38 ± 8.35	0.00 ± 0.00	0.00 ± 0.00	0.08 ± 0.11	*p* = 0.121
Maximum air temperature (°C)	27.82 ± 4.02a	18.55 ± 1.77ab	14.35 ± 5.97b	25.86 ± 3.91ab	*p* = 0.005
Minimum air temperature (°C)	17.6 ± 3.39a	9.3 ± 2.69ab	7.3 ± 2.88b	14.02 ± 1.73ab	*p* = 0.003
Cumulative air temperature (°C)	204.65 ± 105.08	147.70 ± 38.59	60.93 ± 51.10	165.36 ± 91.24	*p* = 0.076
Maximum accumulated radiation (W/m^2^)	43.87 ± 45.47	99.39 ± 79.60	72.75 ± 73.88	86.09 ± 91.68	*p* = 0.995
Accumulated radiation (W/m^2^)	195.24 ± 105.38	177.23 ± 30.48	180.22 ± 133.60	202.70 ± 133.06	*p* = 0.589
Superficial soil temperature (°C)	23.44 ± 1.78a	10.01 ± 0.87b	10.10 ± 2.09b	20.80 ± 1.88a	*p* = 0.002
Lower soil temperature (°C)	23.53 ± 1.34a	11.37 ± 0.31bc	10.32 ± 0.00c	20.41 ± 1.14b	*p* = 0.001
Relative humidity (%)	87.14 ± 8.12	80.55 ± 8.72	87.28 ± 13.66	88.15 ± 6.26	*p* = 0.652
Soil moisture (%)	17.50 ± 4.15a	18.53 ± 0.42ab	19.89 ± 0.50a	8.38 ± 0.41b	*p* = 0.010
Relative humidity during assay (%)	78.84 ± 17.98	82.53 ± 17.04	72.55 ± 20.73	67.00 ± 23.33	-
Soil moisture during assay (%)	17.31 ± 4.35	19.65 ± 0.41	20.40 ± 1.72	8.50 ± 0.32	-

Values are presented as mean ± standard deviation. For precipitation, erosivity, temperature, radiation, relative humidity and soil moisture during events, statistics were calculated from individual rainfall events occurring within each seasonal assay. Variables designated as “during assay” were calculated from continuous meteorological measurements recorded throughout the entire 30-day experimental period. Standard deviations for these variables represent temporal variability during the assay and were not included in between-season statistical comparisons. Different superscript letters indicate significant differences among seasons (*p* < 0.05). I30, maximum rainfall intensity in 30 min; E·I30, rainfall erosivity index (product of total storm energy and maximum 30-min intensity); mm, millimeters; mm min^−1^, millimeters per minute; MJ·mm/ha·h, megajoules millimeters per hectare per hour; °C, degrees Celsius; W/m^2^, watts per square meter; %, percentage.

The number of rainfall events varied among seasons, with summer presenting the highest frequency of precipitation events (*n* = 10), followed by spring (*n* = 5), winter (*n* = 4), and autumn (*n* = 2).

Significant seasonal differences were detected for daily and cumulative precipitation (*p* = 0.043 and 0.003, respectively), I30 (*p* = 0.006), maximum and minimum air temperature (*p* = 0.005 and 0.003, respectively), superficial soil temperature (*p* = 0.002), lower soil temperature (*p* < 0.001), and soil moisture (*p* = 0.010).

Summer exhibited the highest mean daily and cumulative precipitation (18.05 ± 12.47, 119.99 ± 56.64 mm, respectively) and rainfall intensity (7.87 ± 5.02 mm), together with elevated air temperatures (maximum: 27.82 ± 4.02; minimum: 17.6 ± 3,39) and soil temperatures (superficial: 23.44 ± 1.78 °C; lower: 23.53 ± 1.34 °C). Cumulative precipitation and I30 during summer were significantly higher than during autumn, winter and spring. In contrast, autumn showed the lowest daily and cumulative precipitation occurrence (1.30 ± 0.14; 4.30 ± 0.99 mm, respectively) and reduced rainfall intensity (0.60 ± 0.57 mm), while presenting comparatively elevated solar radiation values (99.40 ± 79.60 W/m^2^).

Winter was characterized by the lowest soil temperatures recorded throughout the study (superficial: 10.10 ± 2.09 °C; lower: 10.32 ± 0.00 °C), which differed significantly from both summer and spring. Air temperatures were also lowest in winter, differing significantly from summer. Soil moisture reached its highest values during winter (19.89 ± 0.50%), with differences compared to spring. During spring, soil moisture was significantly lower than during summer and winter presenting the lowest values of the annual cycle (8.38 ± 0.41%). Soil temperatures and solar radiation increased again during spring. Precipitation increased relative to autumn and winter (without statistically significant differences), although rainfall intensity remained moderate.

### 3.2 Occurrence of OTC in runoff water

OTC was detected in runoff water during all seasonal assays (Figure 1). The highest OTC concentrations were recorded during summer, ranging from 0.20 to 0.26 μg/mL (0.03–0.07% of the initial fortified concentration), with peak values detected on day 17 of the assay. Residues remained detectable at the end of the experimental period, with concentrations of 0.20 μg/mL still observed on day 30. OTC concentrations in summer were significantly higher than those recorded in autumn, winter, and spring, whereas no significant differences were detected among the remaining seasons (*p* < 0.001). During autumn, OTC concentrations ranged between 0.15 and 0.17 μg/mL (0.04–0.05%), with detectable residues also persisting until the final sampling event. In winter, runoff concentrations progressively decreased throughout the assay, from initial values of 0.15–0.20 μg/mL (0.04–0.06%) during early precipitation events to approximately 0.11 μg/mL (0.01%) toward day 22. Spring showed comparatively stable OTC concentrations throughout most of the experimental period, ranging from 0.13 to 0.15 μg/mL (0.02–0.04%) between days 4 and 21, with detectable residues still present during the final sampling events.

**Figure 1:**
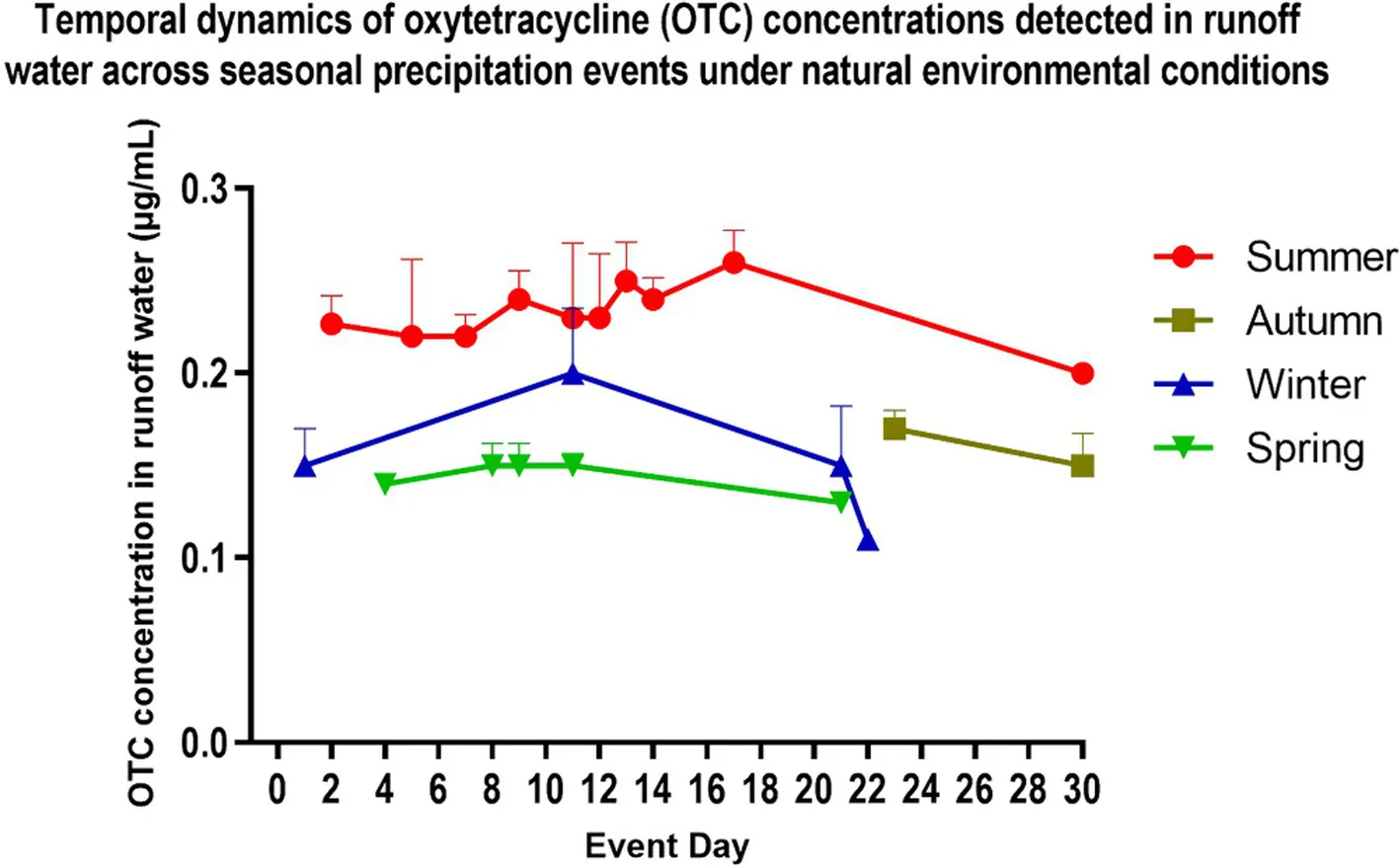
Seasonal oxytetracycline (OTC) concentrations detected in runoff water collected after natural precipitation events during the 30-day environmental exposure assays. Data represent the mean ± SD OTC concentration obtained from the three runoff collectors sampled during each precipitation event day. OTC residues remained detectable throughout all seasonal assays, demonstrating sustained runoff-associated mobilization from swine fecal material under natural environmental conditions.

These findings indicate prolonged environmental persistence and continuous hydrological transport of OTC from fecal deposits under naturally occurring rainfall conditions.

### 3.3 Relationship between OTC occurrence and rainfall events

OTC was detected in runoff water after all recorded rainfall events throughout the experimental period. Marked differences in OTC concentrations were observed according to rainfall characteristics and seasonal environmental conditions (Figure 2).

**Figure 2:**
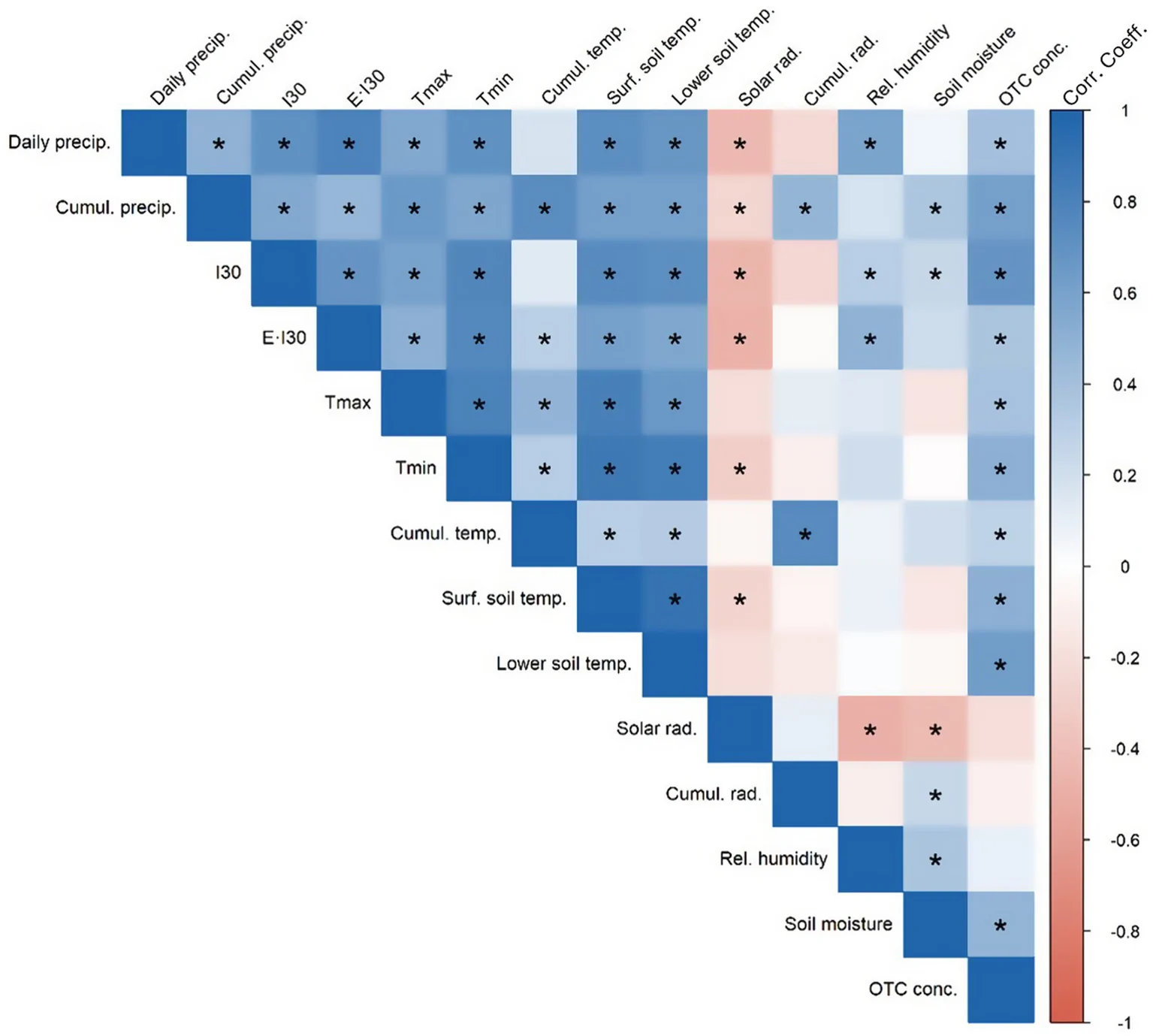
Spearman rank correlation matrix showing associations among oxytetracycline (OTC) concentrations in runoff water and meteorological and hydrological variables. Color intensity reflects the magnitude of the correlation coefficient, with blue indicating positive correlations and red indicating negative correlations. Asterisks (*) indicate statistically significant correlations (*p* < 0.05). Daily precip., daily precipitation; Cumul. precip., cumulative precipitation; I30, maximum 30-min rainfall intensity; E·I30, rainfall erosivity index; Tmax, maximum air temperature; Tmin, minimum air temperature; Cumul. temp., cumulative air temperature; Surf. soil temp., superficial soil temperature; Lower soil temp., lower soil temperature; Solar rad., solar radiation; Cumul. rad., accumulated solar radiation; Rel. humidity, relative humidity; Soil moisture, soil moisture content; OTC conc., oxytetracycline concentration; Corr. Coeff., correlation coefficient.

Higher OTC concentrations were generally associated during precipitation events characterized by elevated rainfall intensity (I30: rs = 0.686, *p* < 0.001) and greater cumulative precipitation (rs = 0.611, *p* < 0.001), particularly during summer assays, when the highest runoff-associated concentrations were detected. Precipitation events with higher erosivity values (E·I30) also coincided with increased OTC concentrations in runoff water (rs = 0.374, *p* = 0.003).

Periods characterized by elevated soil water content, especially during winter assays, were associated with sustained detection of OTC residues throughout the exposure period (rs = 0.477, *p* < 0.001), despite the lower rainfall intensity recorded during this season.

Residues remained detectable even during late precipitation events occurring near the end of the assays, including detections on day 30 during summer and autumn.

Figure 3 illustrates the temporal relationship between OTC concentrations, cumulative precipitation, and rainfall intensity (I30) during runoff events across the four evaluated seasons.

**Figure 3:**
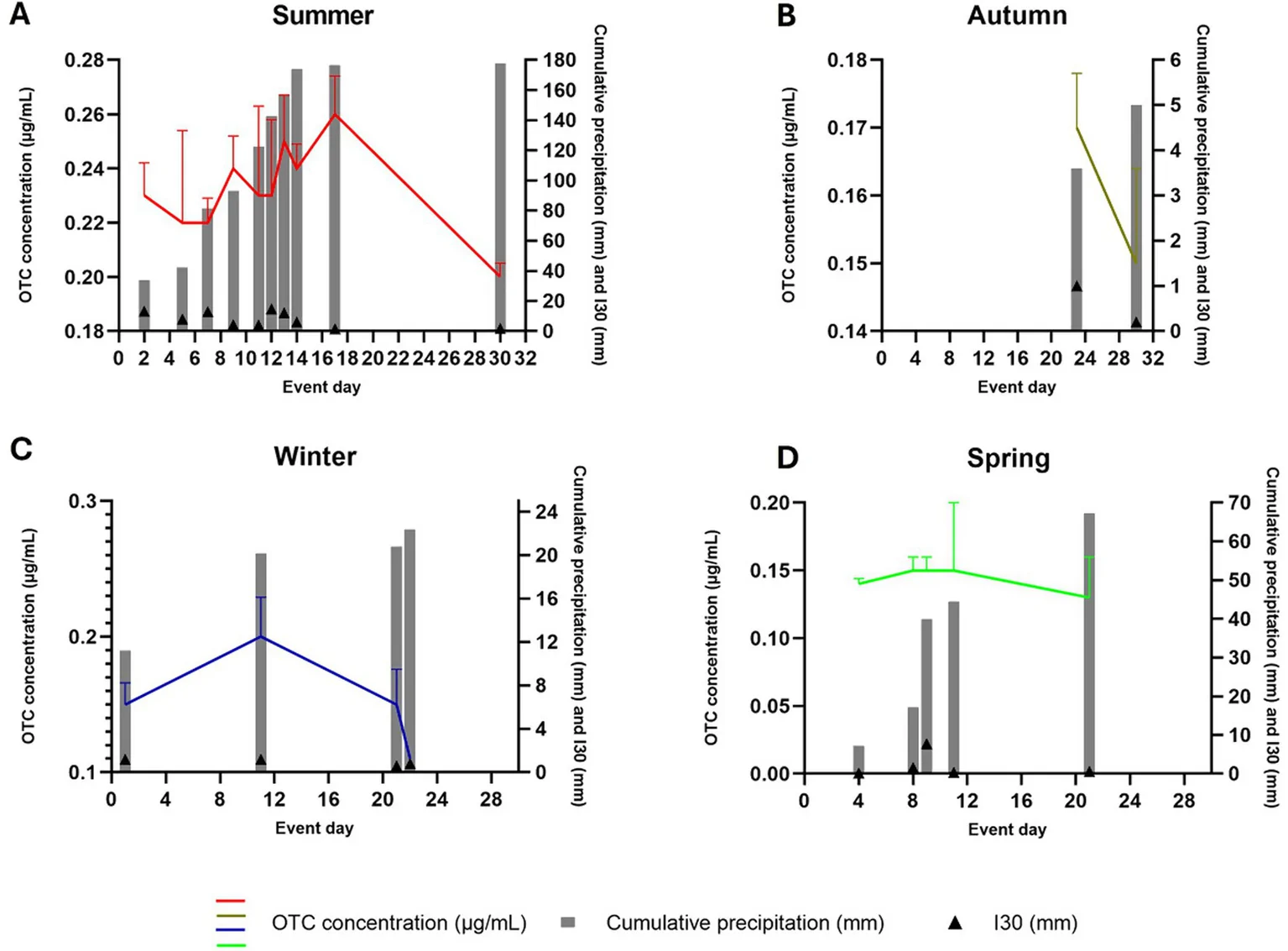
Seasonal relationship between oxytetracycline (OTC) concentrations in runoff water, cumulative precipitation, and rainfall intensity (I30) under natural environmental conditions. OTC concentrations detected in runoff water are shown as lines (μg/mL), cumulative precipitation as bars (mm), and rainfall intensity (I30) as symbols (mm) throughout the experimental period. Values represent the mean OTC concentrations obtained from the three runoff collectors sampled during each precipitation event. Panels correspond to: **(A)** summer, **(B)** winter, **(C)** autumn, and **(D)** spring.

### 3.4 Sediment generation and runoff-associated particulate transport

Given that sediment was collected by pooling the runoff from all precipitation events throughout each 30-day experimental period, a single gravimetric value was obtained per collector per season. Accordingly, sediment data are presented descriptively and no inferential comparison among seasons was performed.

Marked seasonal differences were observed in sediment production associated with runoff processes (Figure 4). Sediment masses recovered at the end of each seasonal assay varied among passive collectors within the same experimental period.

**Figure 4:**
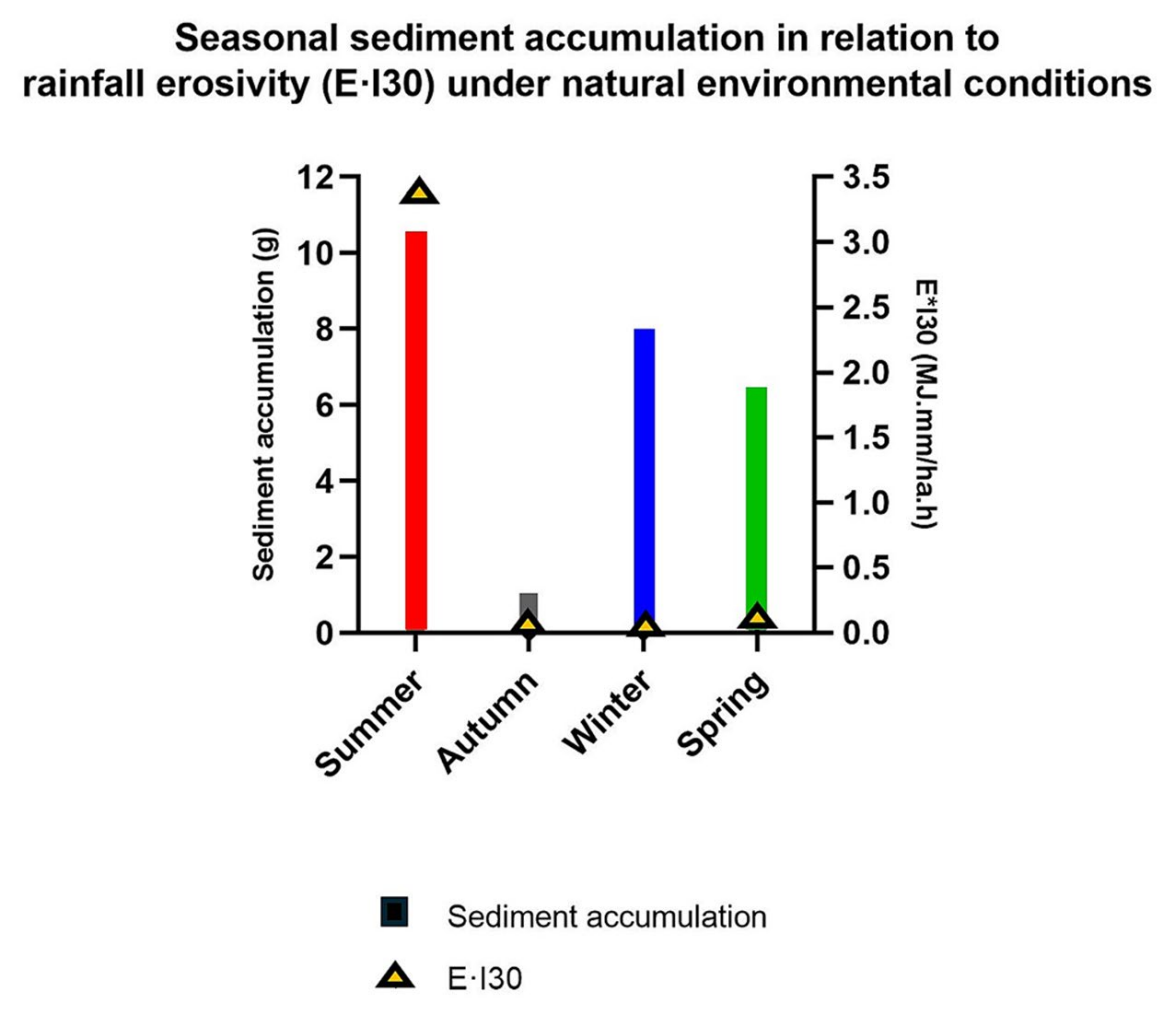
Seasonal sediment accumulation recovered from passive runoff collectors in relation to mean rainfall erosivity (E·I30) during environmental exposure assays. Bars represent the mean sediment mass (g) recovered per season from the three passive runoff collectors, while the line indicates the mean seasonal E·I30 values calculated from recorded precipitation events. Higher sediment accumulation was observed during seasons characterized by greater rainfall erosivity, particularly during summer assays.

The highest sediment accumulation was observed during summer, with total recovered sediment ranging from approximately 6.7 to 16.1 g per collector. In contrast, autumn showed the lowest sediment production values (0.5–1.9 g), whereas intermediate sediment accumulation was recorded during winter (4.8–11.6 g) and spring (5.2–8.8 g).

Higher sediment recovery values were recorded during seasons presenting elevated precipitation intensity (I30) and erosivity values (E·I30), particularly during summer assays. Lower sediment recovery was observed during autumn, when reduced rainfall occurrence and lower erosivity values were recorded.

### 3.5 Edaphic bacterial counts

#### 3.5.1 Effects of OTC exposure and season on culturable aerobic mesophilic bacteria

Culturable aerobic mesophilic bacterial counts ranged from 7.66 × 10^6^ to 1.86 × 10^8^ CFU g^−1^ throughout the experimental period (Figure 5). OTC-treated soils showed significantly higher bacterial counts than control soils (*p* = 0.021).

**Figure 5:**
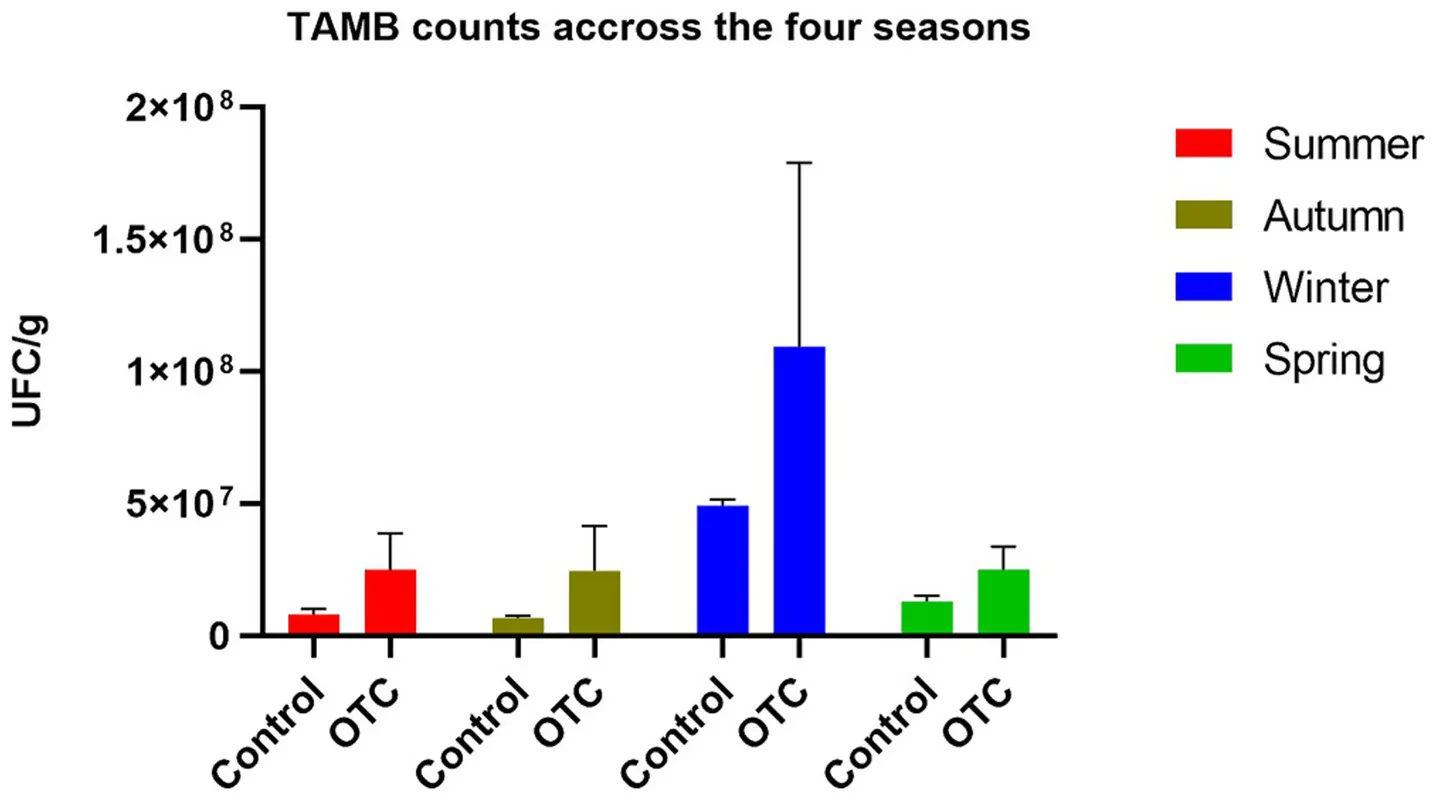
Culturable aerobic mesophilic bacterial counts (CFU g^−1^) in control and OTC-exposed soils during seasonal environmental exposure assays. Values represent mean ± SD obtained from soil samples collected during each seasonal assay. Different lowercase letters indicate significant differences among seasons, whereas asterisks indicate significant differences between control and OTC-treated soils (*p* < 0.05).

Season also significantly affected bacterial abundance (*p* = 0.001). The highest bacterial counts were observed during winter, with values reaching up to 1.86 × 10^8^ CFU g^−1^, whereas lower counts were generally recorded during autumn and spring. In contrast, no significant interaction between treatment and season was observed (*p* = 0.335), indicating that the effect of OTC exposure on culturable bacterial populations remained relatively consistent across seasonal assays.

### 3.6 Modeling of environmental variables associated with OTC concentrations and bacterial responses

#### 3.6.1 Regression models for OTC concentrations in runoff water

For OTC concentrations detected in runoff water, the model including cumulative precipitation, cumulative air temperature during the sampling period, maximum air temperature, and season showed the lowest AICc value (−281.4), although several competing models were also identified (ΔAICc<2).

Cumulative precipitation exerted a significant positive effect on OTC concentrations (*β* = 5.72 × 10^−4^, *p* < 0.001), whereas cumulative air temperature showed a significant negative effect (*β* = −2.60 × 10^−4^, *p* < 0.001).

Regarding seasonal effects, summer (*β* = 7.41 × 10^−2^, *p* < 0.001) and autumn (*β* = 4.34 × 10^−2^, *p* = 0.004) exhibited significantly higher OTC concentrations compared with winter, while spring did not differ significantly from winter (*β* = 2.19 × 10^−2^, *p* = 0.106). Maximum air temperature was retained in competing models but did not show a significant effect in the selected model (*β* = −1.23 × 10^−3^, *p* = 0.123).

#### 3.6.2 Regression models for edaphic bacterial counts

For edaphic bacterial counts, several competing models showed similar explanatory performance (AICc = 898 ± 1.5). The selected model included cumulative precipitation, experimental group (control vs. OTC-exposed soils), average relative humidity during the assay, and average lower-soil temperature during the experimental period.

All variables included in the model showed significant effects on bacterial abundance. Cumulative precipitation was positively associated with culturable bacterial counts (*β* = 1.35 × 10^6^, *p* < 0.001), whereas average relative humidity (*β* = −7.03 × 10^6^, *p* = 0.001) and lower-soil temperature (*β* = −2.03 × 10^7^, *p* < 0.001) were negatively associated with bacterial abundance. OTC-exposed soils exhibited significantly higher bacterial counts than control soils (*β* = 2.53 × 10^7^, *p* = 0.028).

### 3.7 Environmental risk assessment and resistance-selection potential

#### 3.7.1 Potential selective pressure under environmental exposure conditions

All OTC concentrations detected in runoff water exceeded the adopted PNEC_MIC_ value of 0.5 μg/L proposed for resistance selection in aquatic environments. Measured concentrations ranged from 110 to 270 μg/L.

#### 3.7.2 Risk quotients (RQ) associated with OTC runoff concentrations

Calculated RQ values ranged from 220 to 540 depending on the precipitation event and season. The highest RQ values were observed during summer, whereas the lowest values were recorded during winter. All evaluated precipitation events resulted in RQ values above the high-risk threshold (RQ ≥ 1) (Table 2).

**Table 2:** Seasonal ranges of measured environmental concentrations (MECs), risk quotient (RQ) values, and antimicrobial resistance-selection risk categories for oxytetracycline (OTC) detected in runoff water.

Season	MEC range (μg/L)	RQ range	Risk category
Summer	190–270	380–540	High
Autumn	140–180	280–360	High
Winter	110–240	220–480	High
Spring	130–160	260–320	High

## 4 Discussion

The present study demonstrated that OTC residues originating from swine fecal material can persist under natural environmental conditions and remain continuously available for runoff-associated transport following precipitation events throughout different seasons of the year. The detection of OTC in all runoff samples collected during the experimental assays indicates that livestock fecal deposits may constitute sustained environmental sources of antimicrobial residues capable of reaching adjacent environmental compartments through hydrological transport processes. Similar findings have been reported in manure-amended agricultural systems, where tetracyclines were consistently detected in runoff and drainage waters following precipitation events (51, 52). More recently, Huang et al. (25) demonstrated under field conditions that tetracycline antibiotics applied through livestock manure can be mobilized from agricultural soils into surrounding water bodies through rainfall-driven runoff processes, reinforcing the environmental relevance of these dissemination pathways. Field studies have also detected OTC in agricultural irrigation return flows and runoff waters from livestock-impacted systems, confirming the occurrence of tetracycline dissemination under real agricultural conditions (24, 25). The concentrations detected in runoff water throughout the present study were consistently above the analytical quantification limit of the applied HPLC-UV method, supporting the suitability of this analytical approach for monitoring environmentally relevant OTC residues under field conditions. Similar UV-based chromatographic methodologies have previously been successfully applied for the determination of veterinary antibiotics, including oxytetracycline, in environmental water matrices (53, 54).

Seasonal meteorological variability strongly influenced runoff dynamics, sediment generation, and OTC occurrence patterns. Summer conditions, characterized by elevated precipitation intensity, greater rainfall frequency, and increased erosivity values, were associated with the highest OTC concentrations detected in runoff water and the greatest sediment production. These findings agree with previous studies indicating that intense precipitation events enhance contaminant mobilization from agricultural soils and manure-associated matrices through increased runoff generation and particulate transport processes (52, 55). Similar runoff-associated mobilization processes have also been described for other veterinary antimicrobial classes, including sulfonamides and macrolides, reinforcing the role of erosive precipitation and hydrological transport as generalized mechanisms contributing to the environmental dissemination of livestock-derived pharmaceuticals (55, 56). In contrast, autumn exhibited comparatively stable environmental conditions with limited rainfall occurrence and reduced erosive dynamics, whereas winter was characterized by cold and humid conditions potentially favoring OTC persistence within the soil-feces matrix. Previous field studies have similarly reported enhanced persistence and transport of tetracycline residues under cold environmental conditions. Dolliver and Gupta (51) observed that more than 90% of antibiotic detections and losses from manure-amended soils occurred during the non-growing season, which the authors attributed to reduced degradation under low-temperature conditions. Similarly, Kim et al. (16) demonstrated that OTC biodegradation rates decrease substantially under low-temperature conditions, resulting in longer persistence times than those observed at warmer temperatures. These observations support the hypothesis that winter environmental conditions may prolong OTC persistence and maintain environmentally available residue pools over extended periods.

Although rainfall intensity and erosivity parameters (I30 and E·I30) were significant positive associations with OTC concentrations in runoff water (Spearman correlation), these variables were not retained within the best-fitting regression models. Instead, cumulative precipitation emerged as the principal predictor of OTC occurrence in runoff water. This finding suggests that OTC mobilization under field conditions may depend more strongly on progressive water accumulation and prolonged surface saturation processes than on isolated high-intensity rainfall events alone. Repeated precipitation events may gradually enhance antibiotic desorption, dissolution, and transport from fecal and soil matrices, sustaining runoff-associated mobilization over time. Although cumulative air temperature showed a positive Spearman correlation with OTC concentrations, this association likely reflects its co-variation with summer seasonal conditions rather than a direct causal relationship. When cumulative precipitation and season were simultaneously accounted for in the regression model, the partial effect of cumulative air temperature on OTC concentrations was negative, consistent with enhanced hydrolytic and photolytic degradation of OTC under warmer environmental conditions (57, 58). This finding highlights the importance of multivariate approaches for disentangling the independent contributions of correlated environmental predictors.

Furthermore, while increased water availability may facilitate certain hydrolytic processes, sustained soil moisture can simultaneously maintain dissolved and mobilizable OTC fractions within surface environmental matrices, potentially prolonging runoff-associated transport under field conditions.

Although OTC residues remained detectable throughout the entire exposure period, the mechanisms governing runoff-associated mobilization may have differed between early and late precipitation events. Initial runoff episodes likely mobilized more readily soluble or weakly adsorbed OTC fractions located at the feces-soil interface, whereas subsequent precipitation events may have progressively transported more strongly particle-associated fractions as repeated rainfall enhanced surface saturation and hydrological connectivity (52, 59).

Similar observations were reported by Bair et al. (27), who found that increasing irrigation flow rates did not substantially modify OTC concentrations in runoff water under experimentally saturated conditions, suggesting that antecedent soil moisture and sustained surface water availability may exert greater influence on antibiotic transport than flow intensity alone. In that study, soils were intentionally pre-saturated to maximize runoff generation, further supporting the relevance of hydrological saturation processes in controlling antimicrobial mobilization. Likewise, Hoese et al. (60) reported substantial runoff generation following surface manure application due to reduced infiltration associated with crust formation on manure-treated soils, reinforcing the importance of surface hydrological conditions in determining antimicrobial transport under runoff scenarios. Similarly, Burkhardt et al. (56) observed that manure-amended soils generated substantially greater runoff volumes than untreated soils, likely due to partial surface sealing and reduced infiltration capacity. Such hydrological alterations may further enhance the mobilization of manure-associated antimicrobial residues under natural precipitation conditions.

The comparatively low mobilized fractions observed in the present study are consistent with the strong adsorption affinity of tetracyclines for soil particles, organic matter, and fecal material, which limits immediate displacement while favoring prolonged environmental persistence (9, 61). Previous sorption studies have demonstrated extremely high partition coefficients for OTC in soils and sediments rich in organic matter, supporting the strong retention behavior observed under environmental conditions (62). Similarly, Conde-Cid et al. (63) reported adsorption percentages exceeding 85–100% for tetracyclines in agricultural soils, with minimal desorption rates, reinforcing the concept that only a limited fraction remains immediately available for mobilization. Similar observations were reported by Hoese et al. (60), who found lower runoff transport for chlortetracycline than for tylosin, highlighting the importance of antimicrobial physicochemical properties in determining mobilization patterns. Differences in runoff-associated transport among antimicrobial classes have been strongly linked to their sorption behavior and physicochemical characteristics. Davis et al. (55) demonstrated that tetracyclines and macrolides exhibited greater association with sediment fractions, whereas sulfonamides showed comparatively higher mobility in dissolved runoff phases. These observations further support the interpretation that the strong adsorption affinity of OTC likely favored sediment-associated transport pathways under the present field conditions.

The greater sediment recovery observed during summer, coinciding with the highest I30 and E·I30 values, suggests a potential linkage between erosive runoff processes and OTC dissemination. Because tetracyclines exhibit a well-documented high sorption affinity for particulate material, it is plausible that runoff-associated transport involved both dissolved and sediment-associated fractions under field conditions, as previously demonstrated by Davis et al. (55) and Blackwell et al. (61). However, since OTC concentrations were not directly quantified within the recovered solid sediment matrix in the present study, the specific magnitude of this particle-associated transport pathway remains hypothetical for our experimental setup. Nevertheless, the descriptive overlap between high sediment production and elevated runoff concentrations during erosive seasons (such as summer) aligns with the literature indicating preferential association of tetracyclines with sediment and particulate runoff fractions under erosive transport conditions. Li et al. (23) similarly reported that OTC frequently predominated among tetracycline residues detected in river sediments and surface waters, which the authors associated with its strong adsorption to fine particulate material and sediment fractions.

Interestingly, the percentages of OTC detected in runoff water in the present study (approximately 0.01–0.07% of the initial fortified concentration) were comparable to values previously reported under agricultural runoff conditions. Bair et al. (27) detected less than 0.1% of applied OTC in runoff water from irrigated pasture systems, despite experimental conditions specifically designed to maximize surface transport potential, including soil pre-saturation and continuous manure application along runoff flow paths. Similarly, Huang et al. (25) reported that approximately 0.13% of manure-associated OTC was transported through surface runoff from manure-amended agricultural soils. In contrast to the present study, where OTC-containing fecal material remained superficially exposed as a localized source, their study evaluated runoff from a broader manure-treated soil surface in which manure was incorporated into the upper soil layer. The lower percentages observed in the present study may therefore be partially explained by the smaller contaminated area directly exposed to runoff processes. Similar to the present study, Dolliver and Gupta (51) reported that only small fractions of manure-associated antibiotics were mobilized from agricultural soils (<5% of the applied mass). Likewise, Aga et al. (64) reported prolonged persistence of OTC residues in manure-amended soils, while only trace concentrations were detected in deeper soil layers and no residues were detected in field lysimeters, supporting the concept of low vertical mobility despite prolonged environmental persistence. Collectively, these studies indicate that even relatively small mobilized fractions of tetracyclines may generate environmentally relevant antibiotic concentrations in adjacent aquatic compartments due to the high antimicrobial loads associated with livestock wastes. Consequently, limited transport percentages do not necessarily imply low ecological relevance, particularly for compounds capable of exerting biological and selective effects at subinhibitory environmental concentrations.

The persistence of detectable OTC residues until the end of the 30-day exposure period further supports the environmental stability of tetracyclines under field conditions. Residues remained measurable even during late precipitation events occurring near day 30, indicating prolonged availability of mobilizable antibiotic fractions within fecal deposits exposed outdoors. Similar persistence patterns have been described for tetracyclines in agricultural environments, where adsorption interactions reduce immediate degradation while maintaining environmentally available residue pools over extended periods (61). Wang and Yates (65) similarly reported relatively slow OTC degradation rates in agricultural soils, with half-lives ranging between 33 and 56 days at 25 °C, further supporting the prolonged persistence potential of tetracyclines following manure release into environmental matrices. The persistence observed under field conditions may also have been influenced by the physicochemical characteristics of the fecal matrix itself. Organic matter-rich fecal material may reduce light penetration, retain moisture, and provide abundant sorption sites capable of limiting antibiotic dissipation while maintaining environmentally available residue pools over time (9, 57, 61).

Similar persistence patterns have previously been reported under livestock production conditions. De Liguoro et al. (66) observed an oxytetracycline half-life of approximately 30 days in manure from treated calves, with detectable residues still present after five months of manure maturation. Although OTC was not detected in surrounding watercourses in that study, the authors highlighted the prolonged persistence of tetracycline residues within manure-soil systems and their potential environmental relevance.

OTC degradation in environmental matrices is known to be influenced by abiotic factors including temperature, pH, and light exposure, with increasing temperatures generally enhancing hydrolytic degradation processes (57, 58). Under the present field conditions, colder seasonal environments may therefore have reduced OTC dissipation rates, contributing to the prolonged persistence and sustained runoff-associated mobilization observed during winter assays. In addition, photolytic degradation of OTC may become particularly relevant under low-temperature conditions when other dissipation processes are slower (67). Therefore, the reduced solar radiation recorded during winter in the present study may have further contributed to maintaining environmentally available OTC residues within the soil-feces matrix.

OTC residues remained detectable throughout prolonged environmental exposure periods, suggesting the occurrence of continuous subinhibitory exposure scenarios within soil-water interfaces receiving agricultural runoff. Increasing evidence indicates that subinhibitory antibiotic concentrations may promote enrichment of resistant bacterial populations, horizontal gene transfer, and alterations in microbial community structure under environmental conditions (36, 37, 68). From an ecotoxicological perspective, while direct resistance development was not monitored, the sustained occurrence of environmentally available OTC residues suggests a scenario where selective pressure could potentially favor adapted communities within agroecosystems exposed to repeated livestock-associated contamination.

The observed increase in culturable aerobic mesophilic bacterial counts in OTC-exposed soils further supports the potential occurrence of selective microbial responses under environmentally relevant antibiotic exposure. OTC-treated soils consistently exhibited higher bacterial abundances than control soils. This phenomenon, where antibiotic exposure leads to an apparent increase in total culturable counts rather than a decrease, can be attributed to several non-mutually exclusive ecological and physiological mechanisms. First, the sustained subinhibitory concentrations of OTC likely exerted a strong selective pressure that suppressed susceptible strains, facilitating the rapid proliferation and enrichment of intrinsically tolerant or resistant bacterial populations that occupied the vacant ecological niche. Second, low-dose antibiotic exposure can induce hormesis, a biphasic dose–response phenomenon where sub-lethal concentrations can trigger global transcriptional reprogramming and modulate bacterial physiological responses (68), potentially stimulating metabolic activity or culturability in certain adapted strains under specific matrix conditions (69). Additionally, the suppression of specific dominant, sensitive taxa may have reduced overall microbial competition, allowing opportunistic tolerant groups to thrive. It is crucial to highlight that because resistance profiles and molecular determinants were not explicitly characterized in this study, the precise underlying biological mechanisms remain speculative, and these changes do not necessarily reflect a general stimulation of bacterial growth. Furthermore, since both experimental groups received fecal deposits, this bacterial response reflects the specific impact of the antibiotic residue over the baseline nutrient and microbial inputs provided by the fecal matrix itself. Similar observations have been reported in agricultural soils exposed to veterinary antimicrobials, where prolonged low-dose exposure altered microbial community dynamics and favored the persistence of resistant bacterial groups (70, 75).

Seasonal environmental conditions also appeared to modulate bacterial abundance. Winter soils exhibited the highest culturable bacterial counts, likely due to elevated soil moisture, increased relative humidity, and reduced solar radiation, conditions that may decrease desiccation stress and ultraviolet-associated microbial damage. Additionally, lower temperatures and reduced erosive rainfall intensity during winter may have favored greater OTC persistence within the soil microenvironment, potentially prolonging selective pressure on resident microbial communities. Conversely, summer conditions may have promoted increased mobilization of antibiotic residues away from the soil surface through enhanced runoff generation and sediment transport.

Regression analyses further supported the combined influence of environmental variables and antibiotic exposure on soil bacterial dynamics. The positive association between cumulative precipitation and bacterial abundance suggests that water availability may enhance microbial survival and activity under field conditions. In contrast, the negative association observed with mean relative humidity during the assay and lower-soil temperature may appear counterintuitive but likely reflects seasonal confounding, both variables showed their highest values during winter, the season with the lowest bacterial counts in control soils. The negative partial effect of lower-soil temperature on bacterial abundance is consistent with reduced microbial metabolic activity and slower growth rates under cold conditions. The negative association with relative humidity during the assay may reflect the predominance of winter environmental conditions within the model, where elevated humidity coincided with reduced soil temperatures and lower overall microbial activity. Importantly, OTC exposure remained a significant explanatory factor even after accounting for meteorological conditions, reinforcing the ecological relevance of veterinary antibiotic residues in shaping soil microbial responses.

Although TAMB counts do not provide direct information regarding microbial community composition or resistance profiles, they nevertheless indicate relevant alterations in soil microbial dynamics associated with OTC exposure. Future studies integrating microbial community sequencing, antimicrobial resistance gene (ARG) quantification, and analyses of functional microbial groups (e.g., nitrifying and nitrogen-fixing bacteria) will be necessary to discriminate among the potential mechanisms underlying these responses and to better understand the ecological consequences of long-term antibiotic exposure in agricultural soils.

From an environmental risk perspective, all runoff samples largely exceeded the adopted oxytetracycline-specific PNEC_MIC_ threshold proposed for antimicrobial resistance selection in aquatic environments (37, 50). Measured OTC concentrations were between 220- and 540-fold higher than the proposed no-effect concentration associated with resistance selection, resulting in consistently high RQ values throughout all seasons and precipitation events evaluated. These findings indicate that agricultural runoff originating from antibiotic-containing fecal material may represent a relevant pathway for the dissemination of antimicrobial residues at concentrations that exceed theoretical selective thresholds. Consequently, this exposure scenario presents a high potential risk for resistance selection within environmental bacterial communities, although actual molecular dissemination remains to be confirmed in field settings.

Similar observations have been reported in other livestock-impacted environments. Hanna et al. (71) detected antibiotic residues across multiple environmental compartments, including wastewater, sediments, manure, soils, and vegetables, and reported that several compounds exceeded resistance-selection thresholds despite comparatively low measured environmental concentrations. These findings further support the concept that the environmental relevance of antibiotic residues should be interpreted in relation to resistance-selection thresholds rather than absolute concentration magnitude alone.

In addition to dissolved-phase transport, runoff-associated sediment displacement may represent an important dissemination pathway for tetracyclines due to their strong adsorption affinity for particulate material (55, 61, 72). Because runoff samples were centrifuged and filtered prior to chromatographic analysis, the quantified concentrations likely represented predominantly dissolved OTC fractions and fine suspended particles, potentially underestimating the total amount of environmentally transported antibiotic.

Nevertheless, an important strength of the present study lies in the evaluation of OTC mobilization under natural environmental conditions across different seasons of the year. Although this approach inherently involves climatic variability among precipitation events, it provides environmentally realistic information regarding OTC persistence, runoff-associated mobilization, sediment transport, and resistance-selection pressure under field exposure scenarios that are difficult to reproduce under controlled laboratory conditions. Moreover, the integration of seasonal meteorological characterization, erosive rainfall dynamics, sediment generation, and soil bacterial responses within the same experimental framework contributes to a more comprehensive understanding of the environmental behavior of veterinary antibiotics in agricultural systems.

Several limitations should nevertheless be considered when interpreting the present findings. Runoff collectors operated as passive accumulation systems under natural precipitation conditions; therefore, measured OTC concentrations represent residual concentrations in retained runoff water following precipitation events rather than total event-integrated contaminant loads. Because overflow likely occurred during intense rainfall events, the present study focused on environmentally relevant occurrence and persistence patterns of concentrations (MECs) rather than quantifying absolute volumetric transport loads. Additionally, chromatographic analyses were performed on centrifuged and filtered runoff samples; therefore, measured concentrations likely underestimated the particle-associated fraction of OTC mobilization associated with larger particulate and sediment fractions.

The study also focused on culturable aerobic mesophilic bacteria and did not evaluate microbial community composition, resistance genes, or specific resistant taxa. Additionally, runoff transport was assessed under natural climatic conditions, which inherently introduced variability among seasons and precipitation events that could not be fully standardized under field conditions. However, this experimental design also represents a major strength of the study, as it provides environmentally realistic information regarding OTC persistence, mobilization, sediment-associated transport, and resistance-selection pressure under naturally occurring exposure scenarios that are difficult to reproduce under controlled laboratory conditions.

From a One Health perspective, the sustained mobilization of OTC residues from swine feces through seasonal runoff events represents a potentially important environmental pathway contributing to antimicrobial exposure across interconnected environmental compartments. The persistence of antibiotic residues in runoff water, their association with sediment transport processes, and the consistently high resistance-selection risk observed throughout all seasons emphasize the importance of integrating environmental microbiological monitoring into livestock waste management strategies in order to mitigate ecotoxicological impacts and reduce environmental antimicrobial resistance selection pressure.

## 5 Conclusion

The present study demonstrated that oxytetracycline originating from swine feces can persist under natural environmental conditions and remain continuously available for runoff-associated mobilization across different seasons of the year. Seasonal meteorological variability strongly influenced OTC occurrence, sediment generation, and bacterial responses, highlighting the importance of hydrological and erosive processes in controlling antibiotic dissemination under field conditions. Importantly, all detected runoff concentrations exceeded resistance-selection thresholds, indicating and indicating a high potential risk for resistance selection in agroecosystems exposed to livestock-derived waste. While direct molecular evidence of resistance development was not assessed, these findings collectively highlight rainfall-driven runoff as a relevant environmental dissemination pathway that maintains antibiotic concentrations well above selective thresholds, reinforcing the need to incorporate environmental monitoring and runoff mitigation strategies into antimicrobial stewardship frameworks under a One Health perspective.

## Data Availability

The original contributions presented in the study are included in the article/Supplementary material, further inquiries can be directed to the corresponding authors.
